# Efficacy of the traditional Chinese medicine, Buyang Huanwu Decoction, at preventing taxane-induced peripheral neuropathy in breast cancer patients: A prospective, randomized, controlled study

**DOI:** 10.1097/MD.0000000000037338

**Published:** 2024-03-01

**Authors:** Fan Luo, Donggui Wan, Jun Liu, Dongmei Chen, Mengqi Yuan, Chenyang Zhang, Qing Liu

**Affiliations:** a Beijing University of Chinese Medicine, Beijing, China; b Department of Integrative Oncology, China-Japan Friendship Hospital, Beijing, China; c Department of General Surgery, China-Japan Friendship Hospital, Beijing, China.

**Keywords:** breast cancer, Buyang Huanwu Decoction, nab-paclitaxel, prevention, taxane-induced peripheral neuropathy (TIPN)

## Abstract

**Background:**

Buyang Huanwu Decoction (BYHWD) is a traditional Chinese prescription, originally derived from Yi Lin Gai Cuo during the Qing Dynasty. This study aimed to evaluate the efficacy and safety of BYHWD in the prevention of taxane-induced peripheral neuropathy (TIPN) in patients with breast cancer.

**Methods:**

This single-center, statistician-blinded, parallel-group, simple randomized, no-treatment controlled study was conducted at the China-Japan Friendship Hospital in Beijing. Sixty breast cancer patients scheduled to receive nab-paclitaxel-based chemotherapy were randomly assigned to either the BYHWD group (N = 30) or the control group (N = 30) using simple randomization procedures. The data analysts were unaware of the treatment allocation. The primary efficacy endpoints were the incidence and severity of TIPN in the 2 groups, assessed using the Common Terminology Criteria for Adverse Events (CTCAE) and Patients’ Neurotoxicity Questionnaire (PNQ). The secondary efficacy endpoint was the score of Functional Assessment of Cancer Therapy-Breast for both groups. The primary safety endpoints were routine blood test results and liver and renal functions. Both groups were subjected to 4 chemotherapy cycles. Efficacy and safety analyses were conducted on an intention-to-treat basis.

**Results:**

The incidence of TIPN in the BYHWD group was 50.0%, which was lower than the 80.0% incidence in the control group (β = −1.881 [95%CI −3.274, −.488]; *P* = .008, adjusted). The probability of TIPN in the BYHWD group was 15.2% of that in the control group, representing a significant reduction in incidence (odds ratio = .152, [95%CI .038, 0.614]; *P* = .008, adjusted). The CTCAE and PNQ grades of the BYHWD group were 1.527 and 1.495 points lower than those of the control group at the same cycle, respectively (CTCAE: β = −1.527 [95%CI −2.522, −.533]; *P* = .003, adjusted; PNQ: β = −1.495 [95%CI −2.501, −.489]; *P* = .004, adjusted, respectively). After treatment, the Functional Assessment of Cancer Therapy-Breast scores in the BYHWD group were significantly better than those in the control group (*P* = .003), especially in the physiological, functional, and additional concerns domains.

**Conclusion:**

Buyang Huanwu decoction (BYHWD) can effectively prevent TIPN and improve the quality of life in patients with breast cancer.

## 1. Introduction

Taxanes such as paclitaxel and its derivatives are widely used in the treatment of breast cancer. Although effective, peripheral neurotoxicity, known as taxane-induced peripheral neuropathy (TIPN), is a common side effect, which occurs in over 60% of patients receiving paclitaxel treatment.^[1]^ TIPN is characterized by symmetrical tingling and numbness in the limbs, often described as a “gloves-socks” distribution.^[2]^ Other symptoms include impaired fine motor skills. Although these symptoms gradually disappear after drug discontinuation, they can persist for months to years in approximately 30% of patients.^[3]^ TIPN is caused by various pathophysiological changes in the sensory and motor peripheral nerve fibers, resulting in difficulties in postural control, such as an unstable gait and increased risk of falls. This can significantly affect patient quality of life.^[4]^ Furthermore, TIPN can lead to prolonged infusion time, dose reduction, or discontinuation of chemotherapy drugs, which negatively affects treatment outcomes and patient quality of life.

Breast cancer has emerged as the most prevalent form of cancer worldwide, leading to an increasing reliance on taxanes to improve survival rates. In neoadjuvant chemotherapy, nanoparticle albumin-bound paclitaxel (nab-paclitaxel) has shown a significant increase in the pathological complete response rate for breast cancer compared to solvent-based paclitaxel.^[5]^ Additionally, the cost-effectiveness of nab-paclitaxel for breast cancer treatment surpasses that of other taxonomic drugs.^[6]^ However, it is important to note that the incidence of TIPN and TIPN above grade 3 was significantly higher with nab-paclitaxel than with solvent-based paclitaxel (73% vs 46%, *P* < .001; 16% vs 5%, *P* < .001).^[7]^

Currently, the American Society of Clinical Oncology does not recommend drugs for the prevention. Duloxetine is recommended for the treatment of neuropathic pain associated with TIPN.^[8]^ The Chinese expert consensus on the diagnosis and treatment of chemotherapy-induced peripheral neuropathy suggests that the use of pressurized or cryogenic gloves can prevent paclitaxel-related TIPN.^[9]^ However, hypothermic prophylaxis can cause discomfort such as freezing of the hands and feet, which may make it difficult for patients to adhere to the treatment regimen. As such, there is an urgent clinical need to explore additional methods and drugs to prevent TIPN.

Buyang Huanwu Decoction (BYHWD) is a traditional Chinese prescription derived from Yi Lin Gai Cuo in the Qing Dynasty. It is commonly used to treat hemiplegia, mouth and eye deviations, and speech difficulties following stroke. A previous meta-analysis demonstrated that BYHWD effectively prevented the development of oxaliplatin-induced peripheral neuropathy, without causing any adverse reactions.^[10]^ Additionally, a randomized controlled study indicated that the application of BYHWD ointment could prevent TIPN and enhance Karnofsky Performance Status scores.^[11]^

Currently, no clinical study has yet investigated the prevention of nab-paclitaxel-related TIPN in patients with breast cancer. To address this knowledge gap, we conducted a prospective randomized controlled clinical trial to examine the preventive effect and safety of BYHWD on nab-paclitaxel-related TIPN in patients with breast cancer. We hypothesized that BYHWD could effectively prevent TIPN and improve the patients’ quality of life while maintaining good safety.

## 2. Methods

### 2.1. Ethics and permissions

This study was reviewed and approved by the China-Japan Friendship Hospital Clinical Research Ethics Committee (2021-61-K34). The research adhered to the principles outlined in the Declaration of Helsinki, and was conducted in accordance with Good Clinical Practice guidelines. All the patients provided written informed consent before participating in the study.

### 2.2. Sample size

A small randomized controlled clinical trial conducted in China demonstrated that the application of BYHWD ointment can reduce the TIPN incidence caused by solvent-based paclitaxel. In this trial, the TIPN incidence was 18.2% in the BYHWD group compared with 34.9% in the control group.^[11]^ Therefore, it was estimated that BYHWD could potentially reduce the TIPN incidence by half. A meta-analysis revealed that the control group, without any intervention, had a 73% TIPN incidence related to nab-paclitaxel.^[7]^ Based on this data, TIPN incidence in the BYHWD group, which received a traditional Chinese medicine (TCM)-based oral intervention, was estimated to be approximately 36.5% (half the incidence in the control group). For the corresponding statistical analysis, the α value was set to 0.025 (one-sided test with statistical superiority), and the power at 0.8. Using version 11.0 of the Power Analysis and Sample Size software, a sample size of 26 was calculated for each group. Considering an estimated dropout rate of 10%, 30 patients were eventually included in each group.

### 2.3. Study design

This study was a single-center, statistician-blinded, parallel-group, simple-randomized, no-treatment controlled clinical trial conducted at the China-Japan Friendship Hospital in Beijing, China. The patient enrollment period was from April 2021 to December 2022. According to the Chinese Society of Clinical Oncology, a chemotherapy regimen that includes nab-paclitaxel typically lasts for 4 to 6 cycles. Therefore, the observation period was set at 4 cycles because symptoms of TIPN usually manifest within the first 2 months of treatment.

### 2.4. Participants

#### 2.4.1. Eligibility criteria.

Patients with a pathological diagnosis of malignant breast cancer who were receiving a chemotherapy regimen based on nab-paclitaxel; aged between 20 and 75 years; diagnosed with either Traditional Chinese Medicine Differentiation for Qi Deficiency and Blood Stasis Syndrome or Blood Stasis Syndrome;^[12]^ with an expected survival time of more than 3 months; Eastern Cooperative Oncology Group performance status of <2 (including level 2); and ability to independently answer the questionnaire were considered eligible.

#### 2.4.2. Exclusion criteria.

Patients were excluded if they had sensory dyskinesia due to brain or any other metastasis, cerebral infarction, or other cerebrovascular diseases; peripheral nervous system diseases caused by other systemic and metabolic diseases (such as severe diabetes); immune diseases such as myasthenia gravis; peripheral neuropathy induced by previous chemotherapy drugs (paclitaxel, platinum, and vinblastine) that had not recovered; heart, liver, or kidney dysfunction; moderate anemia; coagulation dysfunction; thrombocytopenia; or other contraindications to chemotherapy. Patients were also excluded if they were pregnant or lactating, or were treated with a chemotherapy scheme that contained oxaliplatin, cisplatin, and vinblastine.

### 2.5. Randomization and masking

Sixty patients were randomly assigned to 2 parallel groups in a 1:1 ratio. The BYHWD group received Buyang Huanwu Decoction, consisting of the following medications, medicinal parts, and dosages: huangqi (Milkvetch root, root) 30 g, danggui (Chinese angelica, root) 10 g, chuanxiong (Sichuan lovage rhizome, root) 10 g, honghua (Carthamus tinctorius L, drying flower) 10 g, taoren (peach seed, seed) 10 g, dilong (earthworm, body without internal organs) 10 g, and chishao (peony root, root) 10 g. The control group comprised a no-additional treatment control group.

An independent statistician generated a randomization scheme to assign participants to either the BYHWD or the control group using simple randomization procedures. The details of the allocated group were provided on cards placed in sequentially numbered, opaque, sealed envelopes that were maintained in a predetermined location in each ward. Once patients were enrolled and signed an informed consent letter, the corresponding envelopes were opened sequentially by the opening physician. The opening physician then communicated the grouping information to the medical officer of the ward, who grouped the enrolled patients into either the “control group” or the ‘BYHWD group’ based on the cards. Although the researchers and enrolled patients were not blinded as no placebo was assigned to the control group, the data analysts remained blinded.

### 2.6. Intervention

#### 2.6.1. Control group.

During the observation period, patients in the control group who developed TIPN were provided with vitamin B1 and mecobalamin tablets free of charge, following standard guidelines.^[9]^ The specific medication methods were as follows: vitamin B1 tablets, 10 mg per dose, taken 3 times a day; mecobalamin tablets, 0.5 mg per dose, taken 3 times a day; these were administered continuously orally, and if the symptoms of TIPN disappeared during this period, the tablets could be stopped.

#### 2.6.2. BYHWD group.

Patients in the BYHWD group were orally administered BYHWD for a minimum of 2 weeks in each chemotherapy cycle for a total of 4 consecutive cycles, regardless of the occurrence of TIPN. Buyang Huanwu decoction was prepared in the preparation room of the China-Japan Friendship Hospital. Traditional Chinese medicines at each dose of Buyang Huanwu decoction were decocted into 400 ml with water and separated into 2 200 mL bags. The decoction process followed the standards of the Technical Specification for Decocting Traditional Chinese Medicine. Patients were instructed to drink 1 bag every morning and evening after meals, and the decocted Chinese medicine was refrigerated (0–5 °C) for no more than 3 weeks. When TIPN occurred in the BYHWD group, it was treated with vitamin B1 and mecobalamin tablets using the same method as in the control group. BYHWD and Western medicine should be administered at least half an hour apart.

#### 2.6.3. Background treatment.

Clinicians administered nab-paclitaxel chemotherapy to patients based on the guidelines provided by the Chinese Society of Clinical Oncology. The chemotherapy regimens followed a three-week schedule and included single-drug nab-paclitaxel, nab-paclitaxel combined with anthracycline or cyclophosphamide, nab-paclitaxel combined with lobaplatin, and nab-paclitaxel combined with targeted drugs. Chemotherapy included neoadjuvant chemotherapy, adjuvant chemotherapy, and first-line chemotherapy.

### 2.7. Outcomes

#### 2.7.1. Primary outcome measurement.

The primary outcome measures were the incidence and severity of TIPN in both groups. TIPN was evaluated using the Common Terminology Criteria for Adverse Events (CTCAE) and Patient Neurotoxicity Questionnaire (PNQ). Evaluations were conducted once during each chemotherapy cycle, for a total of 4 times. The CTCAE grades peripheral neuropathy from 0 to 5, with a diagnosis of grade 1 or higher TIPN, where a higher grade indicates more severe symptoms. The PNQ is a self-evaluation questionnaire completed by patients to assess sensory (PNQ1) and motor (PNQ2) nerve symptoms. Each symptom was graded from 0 to 4, with 0 indicating no symptoms, and 4 indicating the most severe symptoms.

#### 2.7.2. Secondary outcome measurement.

The secondary outcome measure was the quality of life of breast cancer patients. Assessment was performed using the Functional Assessment of Cancer Therapy-Breast (FACT-B) Scale, which was administered twice: once before treatment and again after the fourth cycle of chemotherapy. The scale evaluates patients in 5 areas: physiological status, social/family status, emotional status, functional status, and additional attention for patients with breast cancer. The forward items were scored directly, and the reverse items were scored in reverse. The scores for each field and the total score were calculated. A higher score indicated a better quality of life.

#### 2.7.3. Safety assessments.

Adverse reactions were assessed using grading standards of common adverse reactions established by the World Health Organization. Safety endpoints were determined by routine blood tests, liver function, and renal function. The Clinical Event Committee was tasked with reviewing serious adverse events.

### 2.8. Statistical analysis

Analyses were conducted using the Statistical Product and Service Solutions (SPSS version 25.0). Baseline characteristics were presented as means with SDs, medians with IQRs, or counts and percentages, and were compared using appropriate statistical tests, such as the *t*, χ^2^, or rank sum tests, depending on the variable. Primary and secondary outcomes were analyzed according to the intention-to-treat principle using longitudinal data analysis techniques, specifically, generalized estimating equation (GEE) analysis. The analysis included all the randomly assigned participants (N = 60). GEE models were used as these do not require complete data, and can be fitted even when individuals are missing observations at some time points.^[13]^ In this trial, GEE models were performed unadjusted and adjusted for various factors, such as age, Eastern Cooperative Oncology Group score, tissue grade, chemotherapy stage, nab-paclitaxel dose, chemotherapy regimen, obesity, hypertension, and hormone receptor status, which may affect the occurrence of TIPN. A series of GEEs were used to analyze the main effects of group (BYHWD group vs control group) and time (baseline, cycle1, cycle2, cycle3, cycle4), as well as the group × time interaction for each outcome variable. All statistical analyses were two-sided, with a significance level of .05.

## 3. Results

### 3.1. Patient characteristics

**Figure 1.**
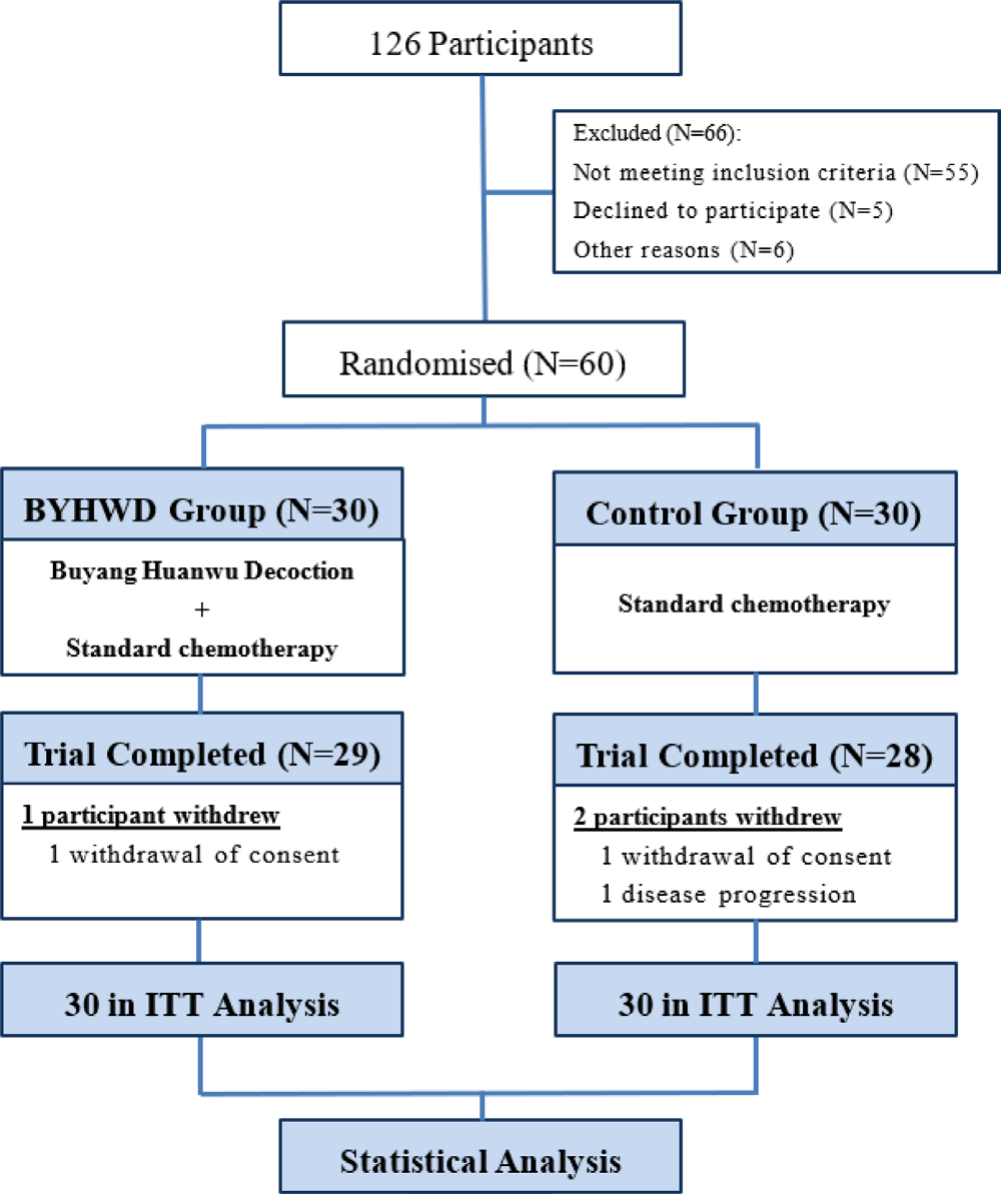
Flow diagram. BYHWD = Buyang Huanwu Decoction, ITT = intention-to-treat.

A total of 126 potential candidates for the study were contacted, and 60 female patients with breast cancer were enrolled from April 2021 to December 2022. Patients were randomly divided into 2 groups: BYHWD (N = 30) and control (N = 30). The patients were followed-up for 4 cycles of chemotherapy. Throughout the trial, 1 participant from the BYHWD group discontinued the intervention because of the voluntary withdrawal of consent, 1 participant from the control group withdrew voluntarily, and 1 participant experienced disease progression. The participant flow diagram is shown in Figure 1.

A review of the literature revealed that decreased levels of estrogen or progesterone,^[14,15]^ older age (55 or 58 years),^[16]^ obesity (body mass index ≥ 30),^[17]^ hypertension,^[16,18]^ and pre-chemotherapy anxiety^[19,20]^ contribute to the occurrence and development of TIPN. As a result, these predictive factors were considered the baseline characteristics of the patients in this trial. Table 1 shows the baseline characteristics, indicating no statistically significant differences in baseline data between the 2 groups.

**Table 1:** Patient characteristics at baseline.

	BYHWD group (N = 30)	Control group (N = 30)	*P* *
Age, n (%)			.30*
<55y	11 (36.7)	14 (46.7)	
≥55y	19 (63.3)	16 (53.3)	
Age, n (%)			.50*
<58y	18 (60.0)	17 (56.7)	
≥58y	12 (40.0)	13 (43.3)	
BMI, n (%)			1.00*
<27.5	27 (90.0)	28 (93.3)	
≥27.5	3 (10.0)	2 (6.7)	
Hypertension, n (%)	4 (13.3)	2 (6.7)	.67*
Organization grade, n (%)			.08**
I	1 (3.3)	1 (3.3)	
II	21 (70.0)	14 (46.7)	
III	8 (26.7)	15 (50.0)	
ECOG score, n (%)			.95**
0	10 (33.3)	9 (30.0)	
1	17 (56.7)	19 (63.3)	
2	3 (10.0)	2 (6.7)	
Hormone receptor state, n (%)			1.00*
Negative	13 (43.3)	13 (43.3)	
Positive	17 (56.7)	17 (56.7)	
Chemotherapy stage, n (%)			.31*
Neoadjuvant	6 (20.0)	2 (6.7)	
Adjuvant	11 (36.7)	14 (46.7)	
First-line and above	13 (43.3)	14 (46.7)	
Chemotherapy regimen, n (%)			.68*
Single-drug nab-paclitaxel	1 (3.3)	3 (10.0)	
nab-paclitaxel with anthracycline or cyclophosphamide	11 (36.7)	13 (43.3)	
nab-paclitaxel combined with carboplatin	11 (36.7)	9 (30.0)	
nab-paclitaxel combined with targeting drugs	7 (23.3)	5 (16.7)	
nab-paclitaxel dose (per cycle), n (%)			.08*
100 mg d1, 200 mg d8	4 (13.3)	5 (16.7)	
200 mg d1, 8	24 (80.0)	17 (56.7)	
320–400 mg d1	2 (6.7)	8 (26.7)	
FACT-B score before treatment, mean (SD)	105.33 (11.55)	101.87 (12.24)	.26***

BMI = body mass index, BYHWD = Buyang Huanwu Decoction, ECOG = Eastern Cooperative Oncology Group, FACT-B = Functional Assessment of Cancer Therapy-Breast, nab-paclitaxel = nanoparticle albumin-bound paclitaxel.

* *P* value was calculated using the chi-square test, or Fisher exact test.

** *P* value was calculated using rank sum tests.

*** *P* value was calculated using independent *t* test.

### 3.2. Efficacy

#### 3.2.1. Primary outcome.

**Figure 2.**
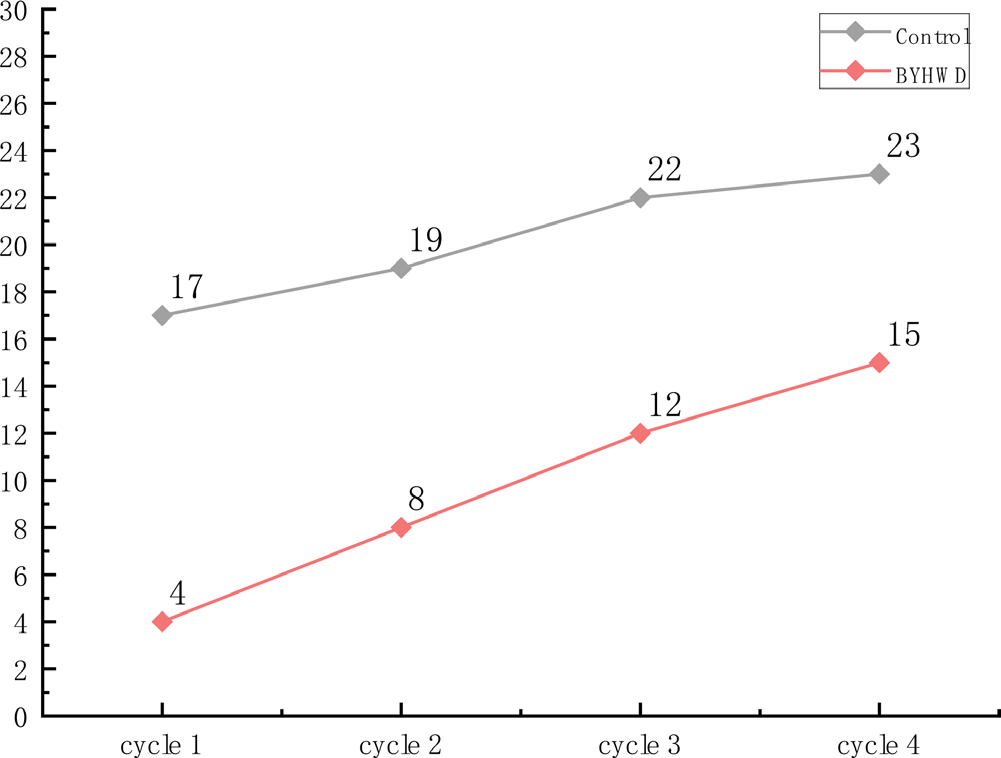
Number of TIPN cases per cycle in the 2 groups. BYHWD = Buyang Huanwu Decoction, TIPN = taxane-induced peripheral neuropathy.

The incidence of TIPN in the BYHWD group (15/30, 50%) was significantly lower than that in the control group (24/30, 80%) (β = −1.881 [95%CI −3.274, −.488]; *P* = .008, adjusted). After excluding the effect of baseline factors, the incidence of TIPN in the BYHWD group was 15.2% of that in the control group, resulting in an 84.8% reduction in the incidence of TIPN (odds ratio = .152, [95%CI .038, 0.614]; *P* = .008, adjusted) compared to the control group. There were also significant differences over time (β = 2.165 [95%CI .913, 3.417]; *P* = .001, adjusted) in the incidence of TIPN, indicating that the probability of developing TIPN in cycle 4 was 8.716 times higher than that in cycle 1 (odds ratio = 8.716, [95%CI 2.491, 30.493]; *P* = .001, adjusted) (Table 2). The group by time interaction effect for the incidence of TIPN was not significant (β = 1.941 [95%CI −.452, 4.280]; *P* = .113; not shown in Table 2). The number of TIPN cases per cycle was significantly lower in the BYHWD group than in the control group (Fig. 2). The number of TIPN cases in the control group in cycle 4 was 23, which differed from the total number of TIPN cases in the control group (24 cases) (Fig. 2). This discrepancy can be explained by the fact that 1 patient in the control group only experienced TIPN onset in cycle 1 (CTCAE level 1, PNQ1 level 1), after which TIPN symptoms disappeared without recurrence.

**Table 2:** Effects of Buyang Huanwu Decoction (BYHWD) intervention and time on incidence of TIPN in the 2 groups.

	Generalized estimation equation model					
	Unadjusted intervention effect			Adjusted intervention effect		
	β (95%CI)	*P* value	OR (95%CI)	β (95%CI)	*P* value	OR (95%CI)
BYHWD group	−1.902 (−3.000, −0.805)	.001	0.149 (0.050, 0.447)	−1.881 (−3.274, −0.488)	.008	0.152 (0.038, 0.614)
Control group	0		1	0		1
Time:Cycle 4	1.591 (0.715, 2.468)	<.001	4.911 (2.045, 11.793)	2.165 (0.913, 3.417)	.001	8.716 (2.491, 30.493)
Time:Cycle 3	1.238 (0.492, 1.983)	.001	3.447 (1.636, 7.264)	1.598 (0.469, 2.727)	.006	4.944 (1.599, 15.292)
Time:Cycle 2	0.572 (0.083, 1.061)	.022	1.772 (1.087, 2.890)	0.576 (−0.303, 1.455)	.199	1.779 (0.739, 4.284)
Time:Cycle 1	0		1	0		1

BYHWD = Buyang Huanwu Decoction, OR = odds ratio, TIPN = taxane-induced peripheral neuropathy.

**Figure 3.**
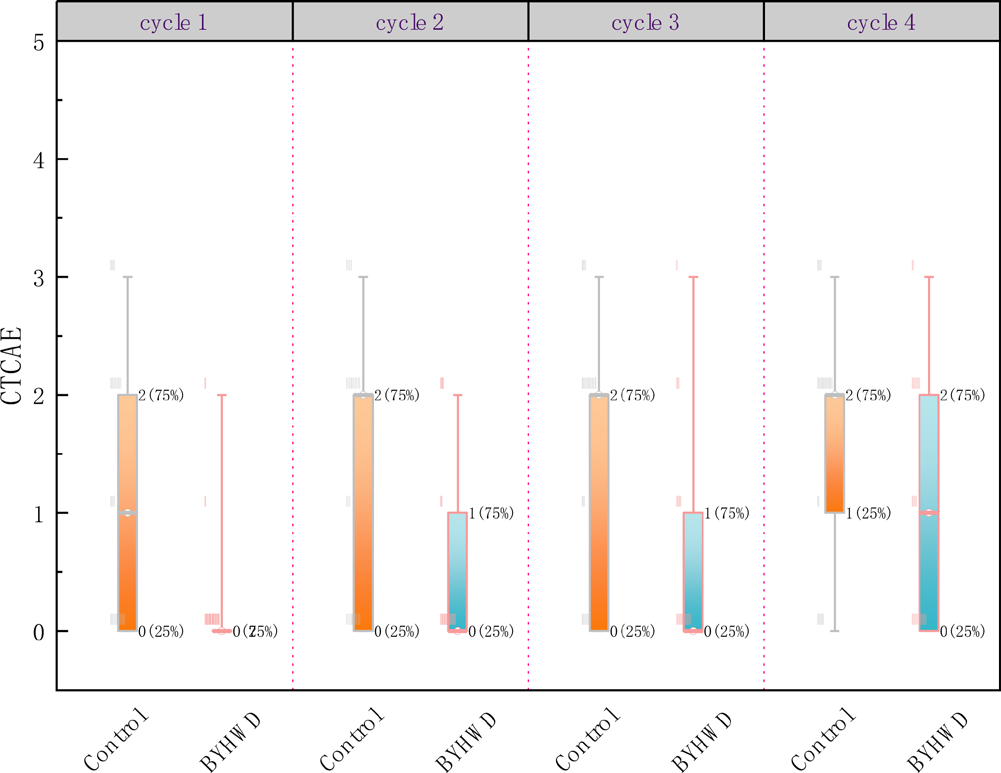
CTCAE classification per cycle in the 2 groups. BYHWD = Buyang Huanwu Decoction, CTCAE = Common Terminology Criteria for Adverse Events.

**Figure 4.**
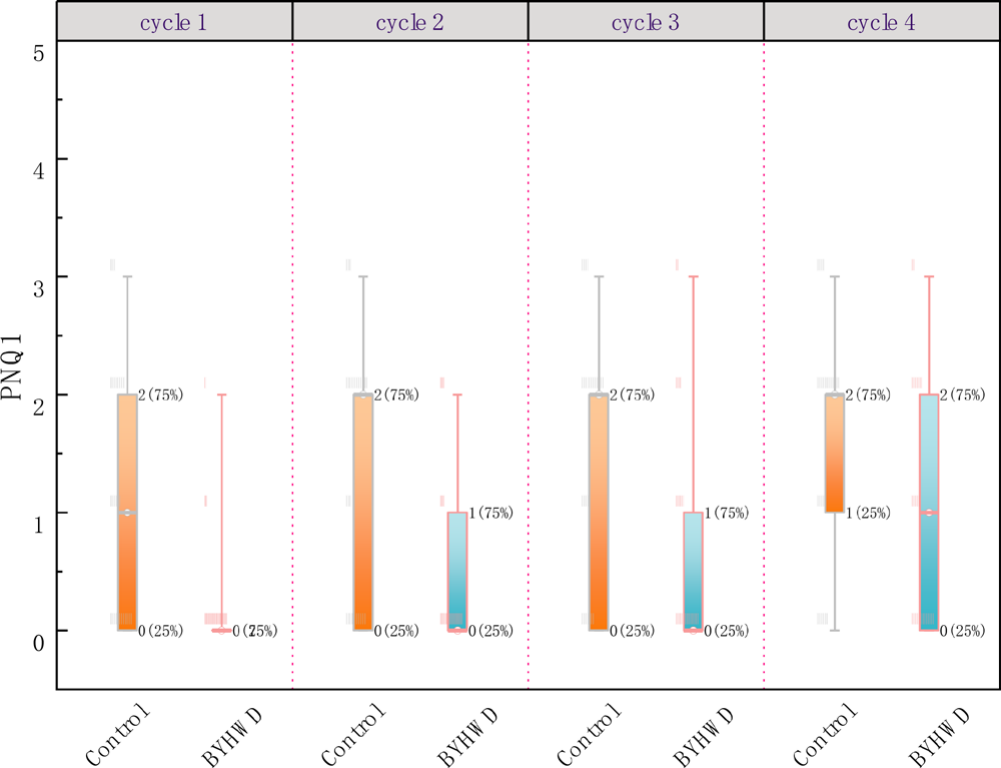
PNQ1 classification per cycle in the 2 groups. BYHWD = Buyang Huanwu Decoction, PNQ = Patients’ Neurotoxicity Questionnaire.

The BYHWD group showed a significant decrease in the CTCAE and PNQ1 classifications compared with the control group. Specifically, the CTCAE and PNQ1 grades of the BYHWD group were 1.527 and 1.495 points lower than those of the control group, respectively (CTCAE: β = −1.527 [95%CI −2.522, −.533]; *P* = .003, adjusted; PNQ: β = −1.495 [95%CI −2.501, −.489]; *P* = .004, adjusted, respectively) (Table 3). The CTCAE and PNQ1 classifications of the 2 groups in each cycle are shown in Figures 3 and 4. Both the groups reported significantly higher grades during the observation period. In cycle 4, participants were likely to be graded 1.789 points and 2.017 points higher for CTCAE and PNQ1, respectively, than in cycle 1 (CTCAE: β = 1.789 [95%CI .969, 2.609]; *P* < .001, adjusted; PNQ: β = 2.017 [95%CI 1.144, 2.890]; *P* < .001, adjusted). The group by time interaction effect for the incidence of TIPN was not significant (β = 1.424 [95%CI −.017, 2.865]; *P* = .053; not shown in Table 3).

**Table 3:** Effects of Buyang Huanwu Decoction (BYHWD) intervention and time on severity of TIPN in the 2 groups.

	Generalized estimation equation model			
	Unadjusted intervention effect		Adjusted intervention effect	
	β (95%CI)	*P* value	β (95%CI)	*P* value
CTCAE grade				
BYHWD group	−1.974 (−2.961, −0.987)	<.001	−1.527 (−2.522, −0.533)	.003
Control group	0		0	
Time:Cycle 4	1.110 (0.048, 1.812)	.002	1.789 (0.969, 2.609)	<.001
Time:Cycle 3	0.862 (0.321, 1.402)	.002	1.408 (0.626, 2.189)	<.001
Time:Cycle 2	0.068 (0.139, 1.077)	.011	0.728 (0.062, 1.393)	.032
Time:Cycle 1	0		0	
PNQ (sensory nerve symptoms) grade				
BYHWD group	−2.235 (−3.778, −0.692)	.005	−1.495 (−2.501, −0.489)	.004
Control group	0		0	
Time:Cycle 4	1.360 (0.686, 2.034)	<.001	2.017 (1.144, 2.890)	<.001
Time:Cycle 3	1.025 (0.443, 1.607)	.001	1.681 (0.847, 2.516)	<.001
Time:Cycle 2	0.623 (0.166, 1.079)	.007	0.871 (0.210, 1.533)	.010
Time:Cycle 1	0		0	

BYHWD = Buyang Huanwu Decoction, CTCAE = Common Terminology Criteria for Adverse Events, PNQ = Patients’ Neurotoxicity Questionnaire, TIPN = taxane-induced peripheral neuropathy.

In the BYHWD group, all participants in the PNQ2 had a grade of 0, indicating that they had no symptoms of motor nerve disorders. In the control group, the incidence of grades 1 and 3 PNQ2 was 10% (3/30), suggesting a low occurrence of motor nerve disorders in TIPN.

#### 3.2.2. Secondary outcome.

There was a significant group-by-time interaction effect for total FACT-B scores between the 2 groups over time. The BYHWD group decreased by 1.76 points, while the control group decreased by 10.24 points over 4 cycles, resulting in a difference of 8.48 points (*P* = .003). The BYHWD group also reported significantly higher total FACT-B scores than the control group (*P* = .009), as well as a significant difference in scores between baseline and cycle 4 (*P* < .001) (Table 4).

**Table 4:** Effects of Buyang Huanwu Decoction (BYHWD) intervention on FACT-B total and each domain scores in the 2 groups.

Variables	BYHWD group		Control group		*P*-value		
	Baseline	Cycle 4	Baseline	Cycle 4	Time	Group	Group × Time
FACT-B total score	105.48 (11.72)	103.72 (10.11)	101.46 (11.95)	91.22 (14.45)	**<.001**	**.009**	**.003**
Physiological domain	24 (22, 26)	23 (21, 24.5)	23 (20, 25.75)	20 (15.25, 22)	**<.001**	.054	**.009**
Social/family domain	22 (18.5, 24)	22 (18.5, 24)	24 (18, 24.75)	23 (18, 24)	.246	.825	.484
Emotional domain	20 (15.5, 22)	20 (15, 22)	19 (14, 22)	16.5 (10.5, 21)	.203	.094	.064
Functional domain	14 (10, 19)	14 (9.5, 16)	13.5 (10, 19)	10.5 (7.25, 15.75)	**<.001**	.576	**.011**
Additional concern	28 (25, 31)	28 (25.5, 31)	27 (26, 29.75)	26 (22, 28.75)	.072	**.013**	**.005**

Significance was assumed when *P* < .05, as shown in bold.

BYHWD = Buyang Huanwu Decoction, FACT-B = Functional Assessment of Cancer Therapy-Breast.

A significant group-by-time interaction effect was further observed for the physiological and functional domains, and additional concerns between the 2 groups over time. In these domains, the BYHWD group showed a smaller decreasing trend than the control group (*P* = .009; *P* = .011; *P* = .005, respectively) (Table 4).

#### 3.2.3. Safety.

This study recorded the most severe adverse reactions in both groups during the follow-up period. The results indicated no significant difference in treatment-related adverse reactions, such as myelosuppression, abnormal liver function, and abnormal renal function, between the 2 groups (*P* > .05).

## 4. Discussion

Our study found that BYHWD, a traditional Chinese herbal medicine, was effective at preventing TIPN. Compared to the control group, BYHWD reduced the incidence of TIPN by 84.8% and decreased its severity according to CTCAE and PNQ1 measurements. Furthermore, BYHWD improves the quality of life of breast cancer patients, particularly in the physiological and functional domains, and addresses additional concerns in patients with breast cancer. This suggests that BYHWD significantly enhances physiological functions and alleviates anxiety and tension among patients with breast cancer. Importantly, treatment with BYHWD for 4 cycles did not cause any adverse events in patients receiving nab-paclitaxel-based chemotherapy.

Our study found that the incidence and severity of TIPN in the control group were consistent with those previously reported. Additionally, we confirmed that BYHWD could prevent paclitaxel injection and improve patients’ physical status.^[11,21]^ In our trial, the incidence of TIPN in the BYHWD group was 50.0%, whereas that in the control group was 80.0%, which is consistent with a previous meta-analysis.^[7]^ The incidence of severe TIPN in the BYHWD group was 6.7% (2 of 30), which was lower than that previously reported, whereas in the control group, it was 23.3% (7 of 30), consistent with the literature.^[7]^ Notably, none of the patients in the BYHWD group showed symptoms of motor nerve disorders, and the incidences of grades 1 and 3 PNQ2 in the control group were only 6.7% and 3.3%, respectively.

The pathogenesis of TIPN has been primarily attributed to ion channel dysfunction,^[22,23]^ mitochondrial dysfunction,^[24]^ neuroinflammatory reaction,^[25,26]^ and other factors. TCM theory suggests that TIPN symptoms are associated with various TCM pathogeneses, including qi deficiency, yang deficiency, and blood stasis. BYHWD is commonly used in the treatment of poststroke conditions, such as hemiplegia, facial asymmetry, and aphasia, as it is believed to benefit qi, promote blood circulation, and improve collateral flow. Previous studies have demonstrated that BYHWD can alleviate paclitaxel-induced peripheral neuralgia in rats by activating spinal cannabinoid type II receptor (CBR2), reducing spinal astrocyte activation, and suppressing the release of inflammatory factors (TNF-α and IL-β).^[27]^ Moreover, BYHWD has shown the potential to promote peripheral nerve repair by enhancing peripheral nerve microcirculation, improving energy metabolism, and boosting cellular immunity.^[28]^ A meta-analysis also indicated that BYHWD was more effective than Western medicine alone in treating peripheral nerve injuries (RR = 1.28 [95%CI 1.19, 1.37], *P* < .001).^[29]^ Other studies have highlighted BYHWD’s ability to facilitate nerve cell repair,^[30,31]^ reduce oxidative stress,^[32–34]^ inhibit inflammatory factors,^[35–37]^ improve microcirculation, and promote angiogenesis.^[33,38,39]^ These findings provide a foundation for further research into the mechanism of action of BYHWD in the prevention and treatment of TIPN. However, it is important to note that this trial had several limitations, including missing data on D-dimer and cytokine levels, which may have introduced bias and led to negative results. Therefore, larger and more rigorous clinical trials are needed to validate the molecular mechanism of BYHWD in preventing and treating TIPN, and to provide more insights for the application and development of Western medicine in TIPN prevention.

This study focused on female patients with breast cancer aged 20 to 75 years who were diagnosed with blood stasis syndrome based on TCM. We included patients at any treatment stage and considered various treatment options, including nab-paclitaxel and excluding paclitaxel, platinum, and vinblastine. These findings have implications in a wide clinical setting.

This study has several limitations. First, it is important to note that this study was conducted at a single center and had a small sample size. Therefore, the conclusions drawn from this study should be further confirmed through larger-scale, multi-center, double-blind studies. Second, there was a certain selection bias in the inclusion of various chemotherapy regimens of nab-paclitaxel in this study, despite the lack of a statistical difference in the dosage of nab-paclitaxel between the 2 groups. Future studies with a multi-center, double-blind, randomized controlled design that strictly stipulates the composition of the chemotherapy regimens are required to address these limitations. Additionally, conducting basic research to further investigate the effect of BYHWD in preventing TIPN and exploring its underlying mechanism would provide a solid foundation for clinical implementation.

## 5. Conclusion

BYHWD has been shown to be effective at preventing TIPN, reducing its severity, and improving the quality of life of patients. However, future studies should aim to achieve greater methodological rigor by including larger sample sizes, longer treatment durations, and placebo controls to validate our findings.

## Acknowledgments

The authors thank all the patients who participated in this study. We would like to thank the researchers of the Clinical Research Data and Project Management Platform of the China-Japan Friendship Hospital for their help.

## Author contributions

**Conceptualization:** Donggui Wan.

**Data curation:** Fan Luo, Qing Liu.

**Investigation:** Mengqi Yuan, Chenyang Zhang.

**Methodology:** Fan Luo, Jun Liu, Dongmei Chen, Qing Liu.

**Project administration:** Qing Liu.

**Supervision:** Donggui Wan.

**Visualization:** Jun Liu, Dongmei Chen.

**Writing – original draft:** Fan Luo.

**Writing – review & editing:** Donggui Wan, Qing Liu.
